# Assessing 72 h vs. 24 h of long-term video-EEG monitoring to confirm the diagnosis of epilepsy: a retrospective observational study

**DOI:** 10.3389/fneur.2023.1281652

**Published:** 2023-10-20

**Authors:** Katharina Timpte, Ulrike Rosenkötter, Philipp Honrath, Yvonne Weber, Stefan Wolking, Jan Heckelmann

**Affiliations:** Department of Epileptology and Neurology, RWTH University Hospital Aachen, Aachen, Germany

**Keywords:** epilepsy monitoring unit, interictal epileptic discharges, interictal epileptiform abnormality, differential diagnosis, antiseizure medication

## Abstract

**Introduction:**

Paroxysmal seizure-like events can be a diagnostic challenge. Inpatient video-electroencephalography (EEG) monitoring (VEM) can be a valuable diagnostic tool, but recommendations for the minimal duration of VEM to confirm or rule out epilepsy are inconsistent. In this study, we aim to determine whether VEM of 48 or 72 h was superior to 24 h.

**Methods:**

In this monocentric, retrospective study, we included 111 patients with paroxysmal, seizure-like events who underwent at least 72 h of VEM. Inclusion criteria were as follows: (1) Preliminary workup was inconclusive; (2) VEM admission occurred to confirm a diagnosis; (3) At discharge, the diagnosis of epilepsy was conclusively established. We analyzed the VEM recordings to determine the exact time point of the first occurrence of epileptic abnormalities (EAs; defined as interictal epileptiform discharges or electrographic seizures). Subgroup analyses were performed for epilepsy types and treatment status.

**Results:**

In our study population, 69.4% (77/111) of patients displayed EAs during VEM. In this group, the first occurrence of EAs was observed within 24 h in 92.2% (71/77) of patients and within 24–72 h in 7.8% (6/77). There was no statistically significant difference in the incidence of EA between medicated and non-medicated patients or between focal, generalized epilepsies and epilepsies of unknown type. Of the 19 recorded spontaneous electroclinical seizures, 6 (31.6%) occurred after 24 h.

**Discussion:**

A VEM of 24 h may be sufficient in the diagnostic workup of paroxysmal seizure-like events under most circumstances. Considering the few cases of first EA in the timeframe between 24 and 72 h, a prolonged VEM may be useful in cases with a high probability of epilepsy or where other strategies like sleep-EEG or ambulatory EEG show inconclusive results. Prolonged VEM increases the chance of recording spontaneous seizures. Our study also highlights a high share of subjects with epilepsy that do not exhibit EAs during 72 h of VEM.

## 1. Introduction

Unclear episodes with transient loss of consciousness or transient behavioral or perceptual alterations are a frequent cause for seeking referral to a specialized neurology service (1). Epileptic seizures are an obvious differential diagnosis in these scenarios. The lifetime prevalence of epilepsy is 1%; it is a common disorder. Establishing the diagnosis of epilepsy can be challenging in cases where patient history, magnetic resonance imaging (MRI), and electroencephalography (EEG) are inconclusive (2–4). Less than 50% of patients with new-onset seizures show MRI abnormalities (5) and, especially in focal epilepsies, the diagnostic yield of routine EEG can be very low (6). To achieve diagnostic certainty, inpatient continuous 24-h EEG or long-term video-EEG monitoring (VEM) on epilepsy monitoring units (EMUs) may be necessary. VEM is considered the gold standard for the differential diagnosis of epilepsy and non-epileptic episodes, e.g., dissociative seizures or syncopes (1, 7–10). It is also useful for seizure type classification, quantification of seizures, and localization of the seizure onset zone during presurgical evaluation (11). Furthermore, VEM is used to estimate seizure recurrence risk after a first unprovoked seizure (12). The evidence about the required length of VEM to make valid diagnostic assumptions is unclear (13). Previous studies pursued this research question in the setting of ambulatory long-term EEG but showed conflicting results (14, 15). One study about ambulatory long-term EEG monitoring concluded that 95% of interictal epileptic discharges (IEDs) that occurred in the 96-h recording had already occurred in the first 48 h (15). In another study, in which 61% of patients carried the diagnosis of epilepsy, IEDs were seen in 26.9% and electrographic seizures in 6% of all patients (14). Moreover, ambulatory long-term EEG may not be available or reimbursed in all clinical settings. In this study, we aimed to compare 24 h with 48 and 72 h of VEM to confirm the diagnosis of epilepsy by assessing the duration until the first occurrence of epileptic abnormalities (EAs), which were defined as interictal epileptiform discharges or electrographic seizures.

## 2. Materials and methods

### 2.1. Study design and patient selection

We performed a retrospective, monocentric observational study. The study was approved by the Ethical Review Board of the Faculty of Medicine at RWTH Aachen and the Center for Translational & Clinical Research Aachen (CTC-A) (EK 479/21 and CTC-A_21_433). Informed consent for the study was waived by the Ethical Review Board of the Faculty of Medicine at RWTH Aachen (EK 479/21) due to the retrospective nature of the study. We screened 620 protocols of patients admitted to the EMU at the University Hospital RWTH Aachen, a tertiary referral center, between November 2017 and November 2021.

The following inclusion criteria were applied: (1) Continuous VEM recording of at least 72 h during admission to the EMU had been done. (2) The indication for EMU admission was to establish a definitive diagnosis due to an inconclusive preliminary diagnostic workup. (3) Upon discharge, the diagnosis of epilepsy had been ascertained. (4) We explicitly included subjects who received antiseizure medication (ASM) at the time of admission but who did not fulfill the ILAE diagnostic criteria for epilepsy (16), e.g., on grounds of patient preference, safety considerations, or a different interpretation of treatment guidelines. This also included subjects with normal findings on routine EEG and MRI and a singular, unprovoked seizure.

**Figure 1:**
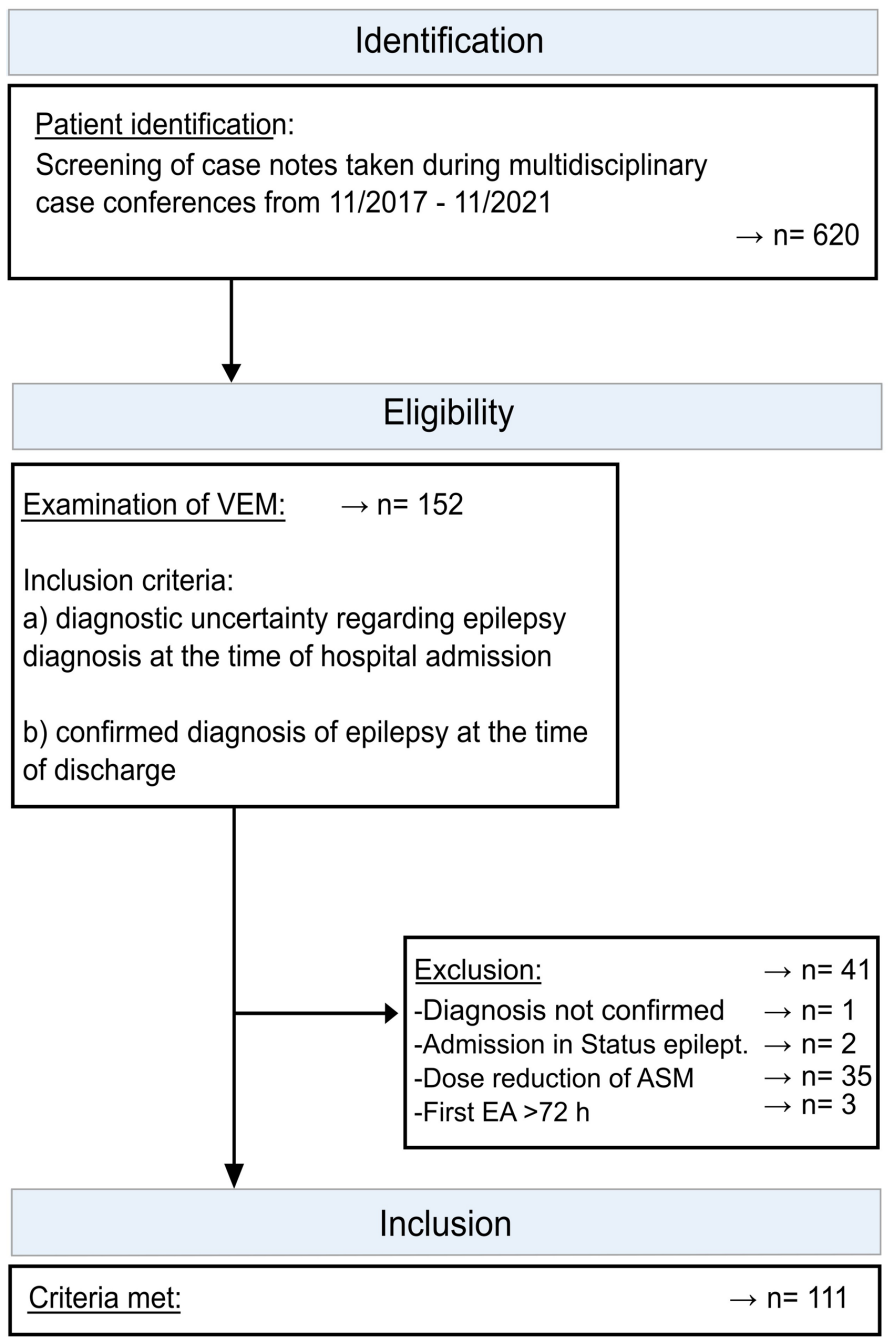
Process of patient selection and formation of subgroups. Flowchart of patient inclusion and reasons for exclusion.

The exclusion criteria were as follows: (1) The diagnosis of epilepsy was revoked after a retrospective assessment of the case; (2) a finding of electrographic status epilepticus at the commencement of VEM recording; (3) ASM reduction or withdrawal during VEM recording; and (4) the first EA after more than 72 h since the start of VEM recording (Figure 1).

### 2.2. Clinical variables

We assessed the following clinical variables: age, sex, epilepsy diagnosis upon discharge, seizure frequency before admission, EEG and MRI results, comorbidities, and ASM upon admission and discharge. We analyzed the time until the occurrence of the first EA and the first seizure in minutes and hours. We employed the current ILAE guidelines for epilepsy classification (16). We performed subgroup analyses according to ASM status: Group 1: no ASM treatment during VEM recording; Group 2: stable ASM regimen. We also performed subgroup analyses for epilepsy types: genetic generalized epilepsy (GGE), focal epilepsy (FE), and unknown epilepsy type.

### 2.3. Assessment of epileptiform abnormalities

Video-EEGs were recorded with the Micromed^®^ recording system. The electrodes were placed using the 10–20 system. The data were manually analyzed in two steps. First, we reviewed existing annotations made by experienced epileptologists in the department within the framework of the primary clinical workup. Second, two independent, experienced epileptologists reevaluated the EEGs for epileptiform abnormalities (EA) that were potentially overlooked during the initial assessment. We measured the time from the start of the recording until the occurrence of the first EA in hours and minutes. EA was defined as either IEDs or rhythmic patterns fulfilling the criteria of electrographic seizures according to the standards of the American Clinical Neurophysiological Society (17). We also determined whether EAs were already detectable during a resting EEG, i.e., an EEG recording of 20 min, including hyperventilation and photostimulation, which was conducted as an integral part of the VEM procedure on the day of admission.

### 2.4. Statistics

Statistical analyses were performed using IBM SPSS Statistics, version 28. To assess statistical differences for various subgroups, we performed the chi-squared tests, Mann–Whitney U tests, and Kruskal-Wallis H tests. An alpha level of *p* < 0.05 was considered statistically significant. Figures were compiled using Inkscape.

## 3. Results

### 3.1. Cohort description

We screened 620 patient files, of which 152 fulfilled the inclusion criteria. In all, 41 patients were excluded in line with the exclusion criteria, resulting in a total of 111 patients (Figure 1). Fifty-nine (53.2%) were female subjects. The age range was 11–81 years, with a mean age of 43.1 (±19.6) years. Nine patients had a diagnosis of GGE, 90 of FE, and 12 of unknown epilepsy type. MRI showed epileptogenic lesions in 34 patients after a thin-layered epilepsy-specific MRI was performed as part of the VEM inpatient stay or MRI images were reevaluated within the framework of the VEM. For further clinical details, see Table 1. At admission, 65 patients had no ASM, and 46 were on stable ASM treatment without changes during the stay.

**Table 1:** Demographic and clinical characteristics of the study participants.

	Total	ASM retention	No ASM
Number of individuals, *n* (%)	111	46 (41.4)	65 (58.6)
Mean age (±SD)	43.1 (±19.6)	41.6 (±20.8)	44.3 (±18.8)
Mean age of epilepsy manifestation (±SD)	38.2 (±20.3)	38.2 (±21.2)	38.1 (±19.8)
Sex, *n* (%)			
Female	59 (53.2)	23/46 (50.0)	36/65 (55.4)
Male	52 (46.8)	23/46 (50.0)	29/65 (44.6)
Seizure frequency on admission, *n* (%)			
<1 per year	39 (35.1)	15/46 (32.6)	24/65 (36.9)
>1 per year	32 (28.8)	18/46 (39.1)	14/65 (21.5)
Monthly	16 (14.4)	5/46 (10.9)	11/65 (16.9)
>1 per month	7 (6.3)	2/46 (4.3)	5/65 (7.7)
Daily	10 (9.0)	4/46 (8.7)	6/65 (9.2)
Frequency not determinable	7 (6.3)	2/46 (4.3)	5/65 (7.7)
Epilepsy diagnoses upon discharge, *n* (%)			
GGE	9 (8.1)	4/46 (8.7)	5/65 (7.7)
FE	90 (81.1)	39/46 (84.8)	51/65 (78.5)
Unknown type	12 (10.8)	3/46 (6.5)	9/65 (13.8)
MRI results, *n* (%)			
Epileptogenic lesion	34 (30.6)	15/46 (32.6)	19/65 (29.2)
No epileptogenic lesion	77 (69.3)	31/46 (67.4)	46/65 (70.8)
EEG results, *n* (%)			
Focal IED	60 (54.1)	23/46 (50.0)	37/65 (56.9)
Generalized IED	11 (9.9)	4/46 (8.7)	7/65 (10.8)
Focal and generalized IED	6 (5.4)	2/46 (4.3)	4/65 (6.2)
Abnormal, no IED	19 (17.1)	8/46 (17.4)	11/65 (16.9)
Normal	15 (13.5)	9/46 (19.6)	6/65 (9.2)

IED, interictal epileptic discharges; EA, epileptiform abnormalities; GGE, genetic generalized epilepsy; FE, focal epilepsy; MRI, magnetic resonance imaging; EEG, electroencephalography; ASM, antiseizure medication. ^*^ The differences between the two groups (continued ASM treatment and no ASM treatment) were not statistically significant (*p* > 0.05) on any of the clinical variables.

### 3.2. Detection of first epileptiform abnormalities

**Figure 2:**
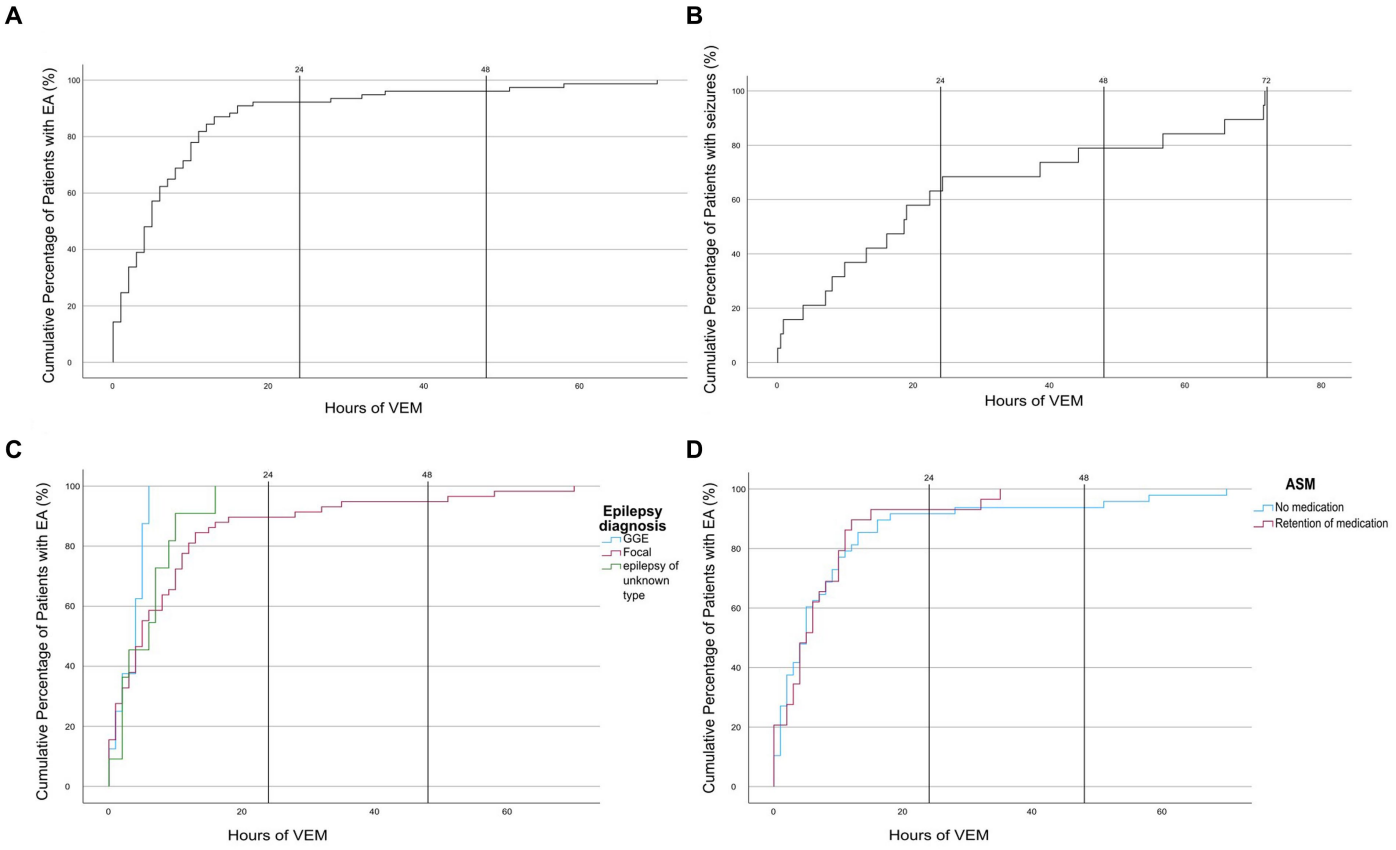
Cumulative time distribution chart (dashed lines at 24 h and 48 h of VEM). **(A)** Time to first EA, **(B)** time to first seizure, **(C)** time to first EA depending on epilepsy diagnosis, and **(D)** time to first EA depending on ASM status. IED, interictal epileptic discharges; EA, epileptiform abnormalities; GGE, genetic generalized epilepsy; ASM, antiseizure medication.

Seventy-seven (69.4%) of the 111 subjects featured EAs during VEM. In 71 of these subjects, EAs were detectable within the first 24 h. In three subjects, the first EA occurred between 24 and 48 h, and in another three subjects, it occurred between 48 and 72 h. The mean time until the detection of the first EA was 8.4 h (SD = 12.5 h). Thus, in 5.4% (6/111) of all subjects, EAs were detected only after 24 h of continuous VEM (Figure 2A).

**Figure 3:**
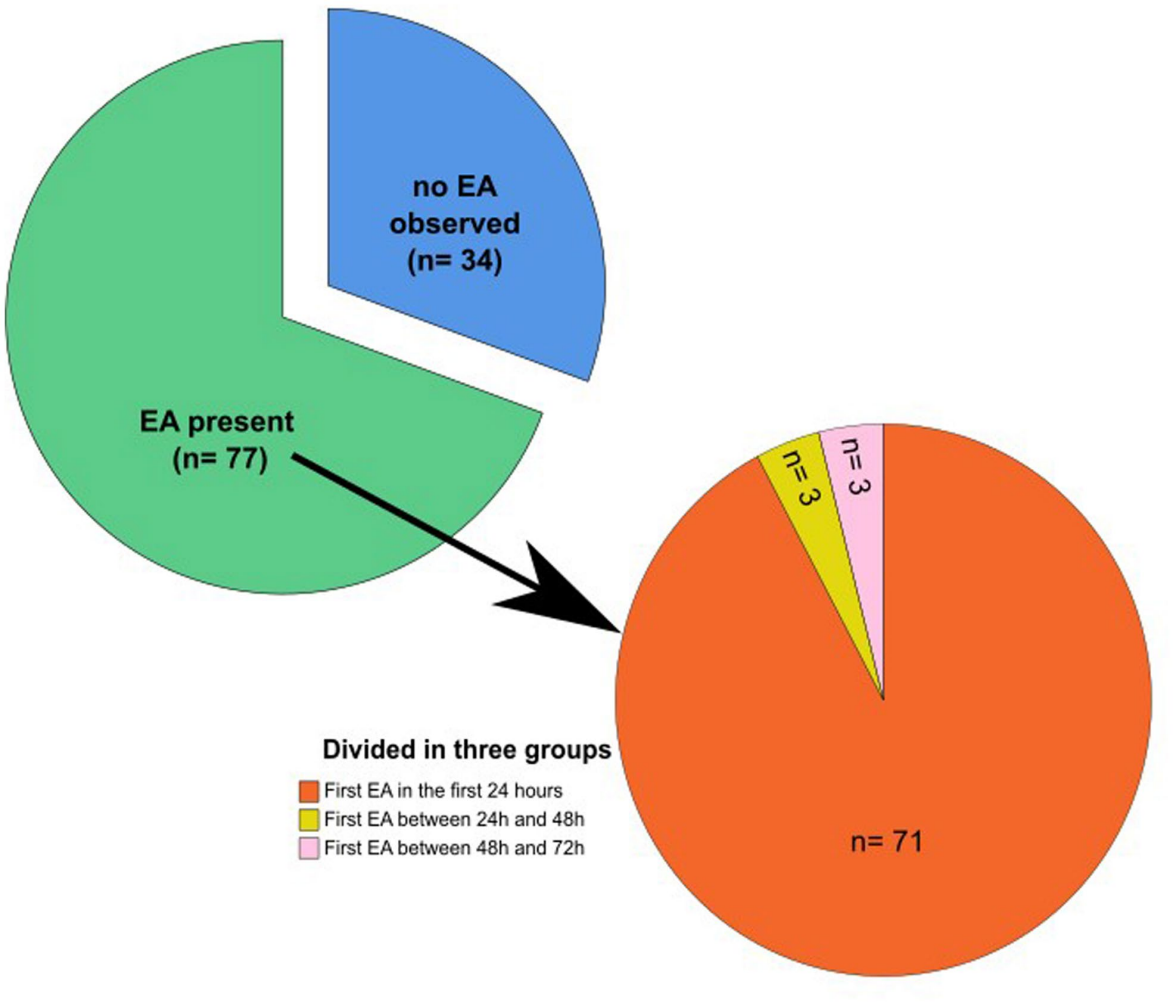
Cohort grouped by time of occurrence of the first EA. Pie chart showing the percentage of patients without any EA and with EA. Second pie chart with subgroup analysis of the EA group: First EA in the timeframe of 0–24, 24–48, and 48–72 h, respectively. EA: epileptiform abnormalities.

In 25 (22.5%) of 111 patients, EAs were already detectable during resting EEG on the first day of VEM. Thirty-four (30.6%) of 111 patients did not show any EA during the recording. For this group, the diagnosis of epilepsy was established based on imaging findings and a reevaluation of available video footage or patient/third-party descriptions of pathognomonic seizure semiology by experienced epileptologists in the VEM ward (Figure 3).

### 3.3. Subgroup analysis by epilepsy type

In GGE, EAs were detected within 24 h in all cases, with an average of m = 3.4 h (SD = 2.1). The average time until the first EA in the FE group was 9.6 h (SD = 14.1 h) and 5.8 h (SD = 4.7 h) in patients with unknown epilepsy type. Group differences were not statistically significant (*Z* = 1,434, *p* = 0.488) (Figure 2C).

### 3.4. Subgroup analysis by ASM treatment group

In all, 46 subjects were on a stable ASM regimen, and 65 subjects had no ASM treatment. In 2 of 46 of the former group and in 4 of 65 of the latter group, the first EA was detected only after 24 h. The mean duration until the first EA among patients with retention of medication was 7.2 h; SD = 8.4 h, and among patients without ASM, it was 9.2 h; SD ±14.5 h (Figure 2D). There was no statistically significant difference between medicated and non-medicated patients (*U* = 692.500, *Z* = −0,037, *p* = 0.971).

### 3.5. Seizure recordings

In 19 of 111 subjects, at least one spontaneous electroclinical seizure was recorded. The average duration until the first seizure was 25.9 (±24.7) h. Notably, 13 (68.4%) of 19 seizures were detected within the first 24 h, 6 (31.6%) of 19 after 24 h (Figure 2B).

## 4. Discussion

We set out to determine whether VEM of 48 or 72 h was superior to 24 h. We found that in most subjects, EAs were detectable within 24 h. A smaller share of subjects seemed to benefit from longer VEM, especially in patients with FE. Prolonged VEM increased the chances of recording spontaneous seizures.

Previous studies on the optimal duration of VEM showed conflicting results. However, the comparability of our results with previous studies is limited. First, the utilization of different EEG techniques (ambulatory vs. inpatient VEM) limits the comparability. Second, previous studies included a broader spectrum of people, including patients with non-epileptic events or undergoing presurgical workups; our study only included patients who were monitored for differential diagnostic workups. In contrast, most previous studies included subjects during presurgical monitoring who underwent drug withdrawal or were limited to certain seizure types (18, 19). The additional detection rate for EA ranged between 3 and 10% for VEM of 72 h vs. 24 h (18, 20–22). A study focusing on IEDs reported a capture rate of 74.6% of the patients with IEDs in VEM within 24 h and of 96.4% after 72 h (23). The differences in methodology render a direct comparison difficult. However, in analogy to our results, most studies conclude that 24 h of VEM appears sufficient for a large share of patients.

We found some advantages for VEM beyond 24 h in our patient population. A non-negligible share of patients showed EA only after 24 h, especially in FE or unknown epilepsy type, corroborating previous findings that showed that IEDs appear earlier in GGE than in FE (24). Interestingly, 25 of the 77 subjects who displayed EAs already did so during our resting EEG assessment. When considering the remaining 52 subjects with EA, the 6 patients with EA after 24 h represent 11.5% of all subjects in which VEM was deterministic for the diagnosis. Approximately a third of spontaneous seizures occurred after 24 h of VEM, even though all patients were on stable ASM or without any ASM. Previous studies reported an average of 2–3 days until seizure occurrence (25, 26); in 35% of patients, seizures occurred after 3 days, and in 7% after more than a week (26). Another study found that 40% of paroxysmal events happened on day 1 of VEM, with a mean VEM duration of 6.9 days (27). In these previous studies, some of the patients underwent ASM withdrawal. We explicitly excluded subjects who underwent drug withdrawal or reduction because reduction speed and steps are usually individually tailored and depend on various aspects such as suspected epilepsy type, estimated risk of generalized seizures, and type of medication, as well as are also adapted to ongoing EEG findings.

Surprisingly, nearly one-third of our subjects did not exhibit any EA during VEM. Previous studies found between 12 and 21% of patients who did not display EAs during VEM, whereby those studies included patients who underwent presurgical workup and ASM withdrawal (18, 23, 28, 29). This could possibly explain the higher share of subjects in our cohort who did not exhibit any EA during VEM. We exclusively included patients who were referred for differential diagnosis and had negative EEG findings, according to the referrers. Furthermore, based on patient and caretaker reports, many of our subjects experienced seizures at a rate of once a year or less. Previous data indicate that patients with <12 seizures per year are less likely to have IEDs during routine EEGs (30).

Our study is limited regarding ASM management since we included patients who were already on stable ASM treatment, although they did not meet ILAE diagnostic criteria for epilepsy at the time of admission. The treatment was usually initiated by the referrer, e.g., on grounds of patient preference, safety considerations, or a different interpretation of treatment guidelines. However, the time until the occurrence of the first EA did not differ between treated and untreated subjects. This could be explained by the large share of FE subjects, in which IED frequency is often not influenced by ASM treatment (31). Due to our clinical setup, we only evaluated patients aged 10 years or older. Since younger children tend to display other types of epilepsy and non-epileptic events, our results cannot be generalized to younger patients.

In conclusion, 24 h of VEM appears to be sufficient to detect EA for most subjects that display EA at all, which should be appraised, especially in resource-limited contexts. However, VEM monitoring for up to 72 h increases the odds of detecting EA in persons with FE and unknown epilepsy types and increases the chance of detecting spontaneous seizures. The complete absence of EA during VEM leaves an unpleasant degree of diagnostic uncertainty. Long-term ambulatory monitoring strategies could help to bridge this diagnostic gap (14, 15, 19). Whether prolonged VEM also provides long-term health or economic effects should be addressed in future studies.

## Data availability statement

The raw data supporting the conclusions of this article will be made available by the authors, without undue reservation.

## Ethics statement

The studies involving humans were approved by Ethical Review Board of the Faculty of Medicine at RWTH Aachen. The studies were conducted in accordance with the local legislation and institutional requirements. Written informed consent for participation was not required from the participants or the participants' legal guardians/next of kin in accordance with the national legislation and institutional requirements.

## Author contributions

KT: Writing – original draft. UR: Writing – review & editing. PH: Writing – review & editing. YW: Writing – review & editing. SW: Writing – review & editing. JH: Writing – review & editing.
